# Translation, Adaption, and Psychometric Testing of the Myanmar Version of the Medical Outcomes Study Social Support Survey for People Living With HIV/AIDS

**DOI:** 10.3389/fpsyg.2021.707142

**Published:** 2021-09-07

**Authors:** Feifei Huang, Wei-Ti Chen, Sai Htun Lin, Min San Tun, Thet Wai Nwe, Yin Thet Nu Oo, Htun Nyunt Oo

**Affiliations:** ^1^School of Nursing, Fujian Medical University, Fuzhou, China; ^2^School of Nursing, University of California Los Angeles, Los Angeles, CA, United States; ^3^Advocacy, Human Right, and Technical Services Department, Secretariat Office, Myanmar Positive Group (MPG), Yangon, Myanmar; ^4^National AIDS Program, Department of Public Health, Ministry of Health and Sports, Nay Pyi Taw, Myanmar; ^5^Health System Research Division, Department of Medical Research, Yangon, Myanmar

**Keywords:** HIV, survey, psychometric adaptation, social support, Myanmar

## Abstract

**Introduction:** Valid and reliable instruments are crucial for measuring perceived social support among people living with HIV (PLHIV). We aimed to investigate the psychometric properties of the English version of the 19-item Medical Outcomes Study Social Support Survey (MOS-SSS) adapted for PLHIV in Myanmar.

**Methods:** Based on a standard cross-cultural procedure, we adapted the MOS-SSS and formed a Myanmar version of the scale (MOS-SSS-M), and then tested its validity and reliability. A sample of 250 eligible PLHIV was collected from a closed Facebook group that included more than 10,000 Myanmars, most of whom were PLHIV.

**Results:** The MOS-SSS-M achieved a Cronbach’s α of 0.82–0.95. Confirmatory factor analysis revealed an acceptable fit index for the four-factor structure. Construct validity was demonstrated by significant association with self-reported HIV stigma and stress levels, and further confirmed by the findings of Rasch analysis.

**Conclusion:** The MOS-SSS-M with a four-factor structure can be used to measure the level and categories of perceived social support among PLHIV in Myanmar.

## Introduction

HIV has become one of the most serious public health issues in Myanmar. In 2018, an estimated 240,000 people were living with HIV(PLHIV) in Myanmar, with 11,000 new infections and 78,000 deaths (Avert, 2019; UNAIDS, 2019). Myanmar has the second highest prevalence of HIV in Southeast Asia, with a prevalence rate of 0.8% (Avert, 2019; UNAIDS, 2019). To help catalyze the country’s rapid scale-up of its HIV prevention, testing, and treatment, the UNAIDS classified Myanmar in 2014 as a “fast-track” country (UNAIDS, 2014). As a result, the antiretroviral therapy (ART) coverage in Myanmar rose to 70% of the country’s PLHIV as of 2018; some studies found that HIV viral load was suppressed in 93% of PLHIV on the treatment (Kyaw et al., 2017; UNAIDS, 2019; Murray et al., 2020). This viral suppression indicates that those PLHIV who received ART would be in good physical health. However, HIV-related psychological distress, such as stress, depression, anxiety, and stigma, still influence the quality of life (QOL) among PLHIV, as those psychosocial needs are rarely addressed (Lazarus et al., 2016; Betancur et al., 2017; Garfin et al., 2019; Olson et al., 2019), especially in Myanmar, which has poor public health resources (UNAIDS, 2019). These HIV-related negative experiences may be countered by establishing strong social support systems (Kim et al., 2017).

Social support, as an important psychosocial factor for QOL (Abrefa-Gyan et al., 2016), refers to the assistance and protection given by others, such as families and friends (Ducharme et al., 1994). Studies have demonstrated that the benefits of social support, such as improved access and adherence to care (Kim et al., 2017), decreased depression (McDowell and Serovich, 2007; Seffren et al., 2018), decreased perceived stigma (Arshi et al., 2020), increased ART adherence (Huynh et al., 2013), increased CD4 counts (Persson et al., 2002), and decreased alcohol use (Hershow et al., 2020), contributes to both mental and physical health among PLHIV. Such support also results in better QOL (Hou et al., 2014; Oetzel et al., 2014; Garfin et al., 2019). Given its positive health impact on PLHIV, social support has been incorporated into clinical interventions in HIV health care (Horvath et al., 2013; Eaton et al., 2019; Garfin et al., 2019). Therefore, a validated psychometric measurement tool is necessary in order to better understand and evaluate social support among PLHIV and promote health-related social support interventions in fighting HIV (Yu et al., 2015).

The Medical Outcomes Study Social Support Survey (MOS-SSS) is one of the most widely used instruments available for measuring social support in PLHIV (Yu et al., 2015; Abrefa-Gyan et al., 2016). The original MOS-SSS, developed by Sherbourne and Stewart (1991), comprised 19 items that measure four categories of social support: (1) Emotional/informational support (the expression of positive affect, empathetic understanding, and encouragement of expressions of feelings/offering of advice, information, guidance, or feedback), (2) Tangible support (provision of material aid or behavioral assistance), (3) Positive social interactions (availability of other persons to do fun things with you), and (4) affectionate support (involving expressions of love and affection).

The MOS-SSS is a brief, multi-dimensional scale developed to assess social support in patients with chronic illness (Sherbourne and Stewart, 1991). With high reliability and validity, the MOS-SSS has been adapted and validated within different cultural contexts and languages: Arabic (Dafaalla et al., 2016), Brazilian (Zucoloto et al., 2019), French (Anderson et al., 2005), Portuguese (Alonso Fachado et al., 2007), Vietnamese (Khuong et al., 2018), Chinese (Yu et al., 2015), and Spanish (Costa Requena et al., 2007). Although the MOS-SSS has been used in a variety of populations, it has not been thoroughly evaluated for use with PLHIV; its applicability to PLHIV needs to be further examined (Yu et al., 2015; Kim et al., 2017).

Similar to PLHIV in other developing countries, PLHIV in Myanmar face challenges in seeking support, as HIV is a highly stigmatized and stressful health condition (Srisorrachatr et al., 2013). As far as we know, no research has validated MOS-SSS in the Myanmar context, and the absence of a social support measure leads to a limited understanding of social support among PLHIV in Myanmar. Therefore, to address this gap, this study aimed to investigate the psychometric properties of a version of the MOS-SSS (MOS-SSS) adapted for PLHIV in Myanmar based on Classical Test Theory (CTT) and Rasch Analysis.

## Materials and Methods

### Design

This study was approved by the relevant institutional review boards. We culturally adapted the MOS-SSS to create a Myanmar version (MOS-SSS-M) and examined the psychometric properties of the scale, which were adherent to the COSMIN (COnsensus-based Standards for the selection of health status Measurement Instruments) checklist (Mokkink et al., 2010a,b).

### Participants

This cross-sectional, descriptive study was conducted in Myanmar from January to May 2020. A sample of 250 eligible PLHIV was recruited from a closed Facebook group that included more than 10,000 Myanmar residents, more than 90% of whom were PLHIV. Other members of the Facebook group were family members of the PLHIV or HIV-related workers who answered members’ questions. The administrators of the Facebook site were healthcare providers and HIV peer group volunteers. By using random sampling methods, the researcher contacted one of every five individuals on the site of the Facebook roster until the targeted sample size was achieved. The screening questions ensured that all participants were at least 18 years of age, were diagnosed with HIV, were able to provide informed consent, and lived within Myanmar. If they agreed to participate and were able to provide informed consent, an individualized survey link was sent to them via the institutional Research Electronic Data Capture (REDCap) system.

### Translation and Adaptation of the MOS-SSS Into the Myanmar Version

We used a version of the 19-item MOS-SSS that was adapted for the Myanmar PLHIV population (the MOS-SSS-M) to evaluate the frequency of four dimensions of social support (Sherbourne and Stewart, 1991). All of the items were rated using a 5-point Likert scale (1 = “none of the time” to 5 = “all of the time”). A higher score indicated a higher level of social support. The Cronbach’s alpha values for the MOS-SSS ranged from 0.91 to 0.98 for the overall scale, and 2-week test-retest reliability as measured by intra-class correlation coefficients ranged from 0.74 to 0.84 (Yu et al., 2015).

Based on Brislin’s (1970) translation model, the MOS-SSS was adapted into the Myanmar context in the following stages: translation, back-translation, comparison, and linguistic adaption (Jones et al., 2001). First, the 19-item MOS-SSS was translated independently from English into Myanmar by a bilingual physician who was providing HIV care in Myanmar. Then, a bilingual researcher back-translated the Myanmar version into English. Later, one member of the research team compared the back-translated English version with the original English scale and found no differences between them. Finally, the MOS-SSS-M was ready for pilot testing.

### Pilot Test of the MOS-SSS-M

The MOS-SSS-M was distributed to 10 PLHIV in Myanmar to evaluate the items’ fluency, readability, and comprehensibility. The interview used structured probes to uncover how PLHIV interpreted items of the MOS-SSS-M to verify its comprehensibility and readability. Example probes included: “Tell me in your own words what this question is asking,” “How did you decide on your answer to this question?” and “What does social support mean to you?” Interviews were audio recorded and transcribed verbatim. None of the participants reported confusion or incomprehension about the items of the scale.

### Psychometric Test of the MOS-SSS-M

We invited 250 PLHIV in Myanmar to complete the MOS-SSS-M (see Supplementary Appendix A); 194 (77.60%) completed the REDCap survey. The reliability and validity of the MOS-SSS-M were examined by CTT and Rasch analysis according to the recommendation in the COSMIN checklist (Terwee et al., 2012).

### Data Collection

All information was collected online through the REDCap system, a web-based survey tool supported by the Clinical and Translational Science Institute (CTSI). Participants completed the 30-min REDCap survey, which included standardized measures to assess demographics, the MOS-SSS-M, the HIV stigma scale (the overall Cronbach’s α in this sample was 0.95; Berger et al., 2001; Steward et al., 2008), and the Perceived Stress Scale for People Living with HIV/AIDS (PSSHIV; the overall Cronbach’s α in this sample was 0.95; Su et al., 2008). The demographic variables included the participant’s age, gender, marital status, ethnicity, educational level, employment status, health insurance, years of living with HIV, and recent CD4 counts and viral load. After completing the survey, participants were reimbursed for their participation.

### Data Analysis

Data analyses were conducted using SPSS 23.0 (IBM, Chicago, IL, United States) and WINSTEPS 3.75.0 (Chicago, IL, United States). Continuous variables were expressed as means and standard deviations (SDs) and categorical variables were expressed as proportions or percentages. Missing data were replaced using mean value substitution; *p* < 0.05 was considered significant.

#### Cross-Cultural Validity

We used the COSMIN checklist with a 4-point scale (Terwee et al., 2012) to measure which of the descriptions on the translated scale adequately reflected the items from the original scale (Mokkink et al., 2010a,b).

#### Structural Validity

We combined the confirmatory factor analysis (CFA) in the CTT and the Rasch analysis to assess structural validity. In the CFA, we examined the best fitting model of the scale using the maximum likelihood method. We evaluated the model’s goodness of fit by using absolute and relative indices (Johnson et al., 2011; Huang et al., 2017), including normed χ^2^ (χ^2^/d*f*) between 1.0 and 3.0, Root Mean Square Error of Approximation (RMSEA; <0.08), Comparative Fit Index (CFI), Tucker-Lewis Index (TLI), and Normed Fit Index (NFI) > 0.9.

In the Rasch analysis, we first examined the unidimensionality assumptions by Principal component analysis (PCA). Then we used the rating scale model (RSM) to assess person separation reliability, person separation index, category probability curves, person-fit statistics, and test information function (TIF; Linacre, 2011; Xu et al., 2018). Pearson’s fit statistics included infit and outfit mean squares, as well as difficult (location) for individual items. TIF was produced from the sum of each item and the information curve in each subscale. Then, the description of each item with the levels of θ could most precisely and reliably gather the necessary information (Baker, 2001). Finally, items were tested for the differential item functioning (DIF) across gender (male and female) and CD4 cell counts (<200, 200–499, and ≥500 cells/mm^3^).

#### Construct Validity

We estimated the convergent validity of the MOS-SSS-M by calculating Pearson’s correlations between each item and its own subscale; the discriminant validity was tested by comparing the item-own subscale correlation with the item-other subscale correlations. The concurrent validity of the MOS-SSS-M was estimated by Pearson’s correlations with the expected significant negative correlation to the HIV stigma scale and the PSSHIV.

#### Internal Consistency

We used Cronbach’s α and corrected item-total correlation to assess the internal consistency of the scale (Johnson et al., 2011).

#### Floor/Ceiling Effect

Floor effects were evaluated by examining the percentage of respondents that obtained the lowest possible scores. Ceiling effects were evaluated by examining the percentage of respondents that got the highest possible scores.

## Results

### Sample Characteristics

The mean age of participants was 28.23 years (*SD* = 17.16); the average years of living with HIV was 6.90 years (*SD* = 6.61). The average recent CD4 count was 667.38 (*SD* = 455.84); the average viral load was 615.00 (*SD* = 1,058.55). The details of the socio-demographic characteristics of the participants are shown in Table 1.

**TABLE 1:** Sociodemographic characteristics of the participants (*N* = 194).

Variables	*N* (%)
**Gender**	
Male	124(63.90%)
Female	68(35.10%)
Transgender	2(1.0%)
**Ethnicity**	
Bamar	152(78.5%)
Chin	2(1.0%)
Kachin	4(2.1%)
Kayin	7(3.7%)
Kayah	1(0.5%)
Mon	9(4.7%)
Rakhine	4(2.1%)
Shan	6(3.1%)
Others*	8(4.2%)
**Marital status**	
Married or steady partner	82(42.2%)
Widowed	20(10.4%)
Separated	9(4.7%)
Divorced	13(6.8%)
Single, never married	70(35.9%)
**Educational level**	
Middle school graduation	26(13.5%)
High school graduation	79(40.9%)
Professional (vocational) training school graduation	2(1.0%)
Some college but no degree	27(14.0%)
College graduation	54(28.0%)
Post-college graduate	5(2.6%)
**Employment status**	
No	39(20.3%)
Part time	42(21.4%)
Full time	113(58.3%)
**Health insurance**	
Not enough	161(83.10%)
Just enough	33(16.90%)
**CD4 cell count**	
<200 cells/mm^3^	58(29.9%)
200–499 cells/mm^3^	39(20.1%)
≥500 cells/mm^3^	97(50.0%)

*Palaung, Islam, Tamil.

### Cross-Cultural Validity

The process of translation and determination of the sample size (≥150) met the requirements of the “good” level in the COSMIN checklist (Terwee et al., 2012). Before the formal survey, we also conducted a pilot test to evaluate the items’ fluency, readability, and comprehensibility; all participants reported a good understanding of each item of the PSSHIV-M.

### Structural Validity

**FIGURE 1:**
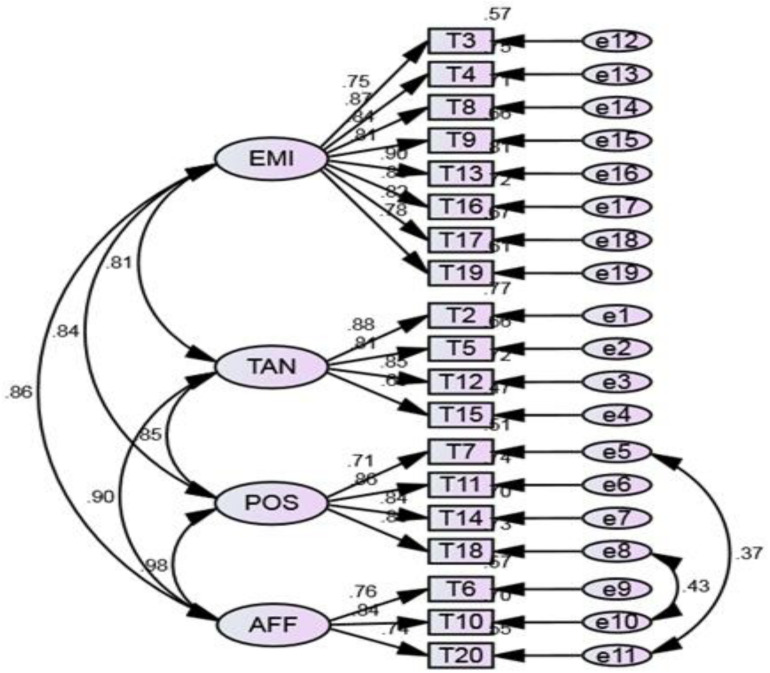
The factor structure of the Myanmar version of the Medical Outcomes Study Social Support Survey. χ^2^/d*f* = 2.120 (*p* = 0.000), Comparative fit index = 0.95, Normed fit index = 0.91, Tucker-Lewis index = 0.94, Root-Mean-Square Error of Approximation=0.07. TAN, tangible support; AFF, affectionate support; POS, positive social interaction; EMI, emotional/informational support.

**FIGURE 2:**
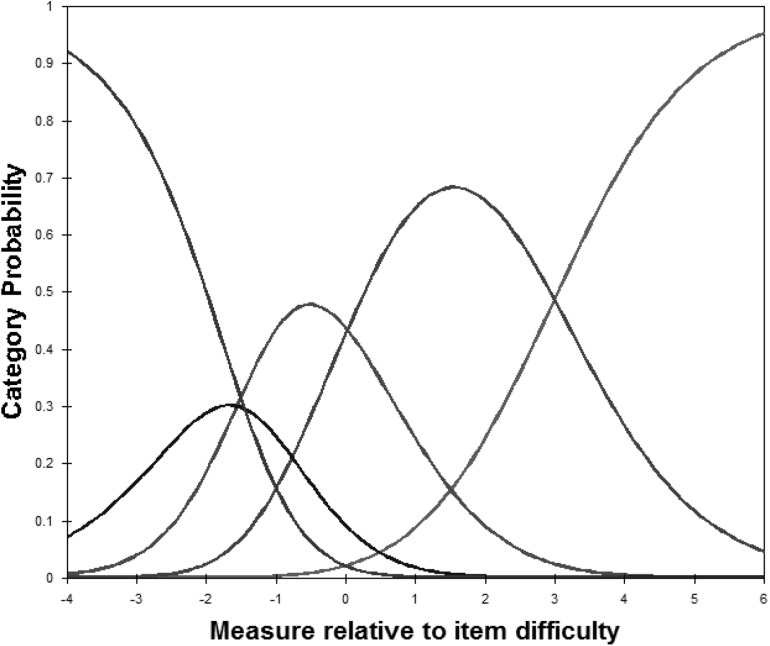
Category probability curves for the Myanmar version of the Medical Outcomes Study Social Support Survey. The five curves from left to right represent 5 response categories (1 = “none of the time,” 2 = “a little of the time,” 3 = “some of the time,” 4 = “most of the time,” to 5 = “all of the time”).

**FIGURE 3:**
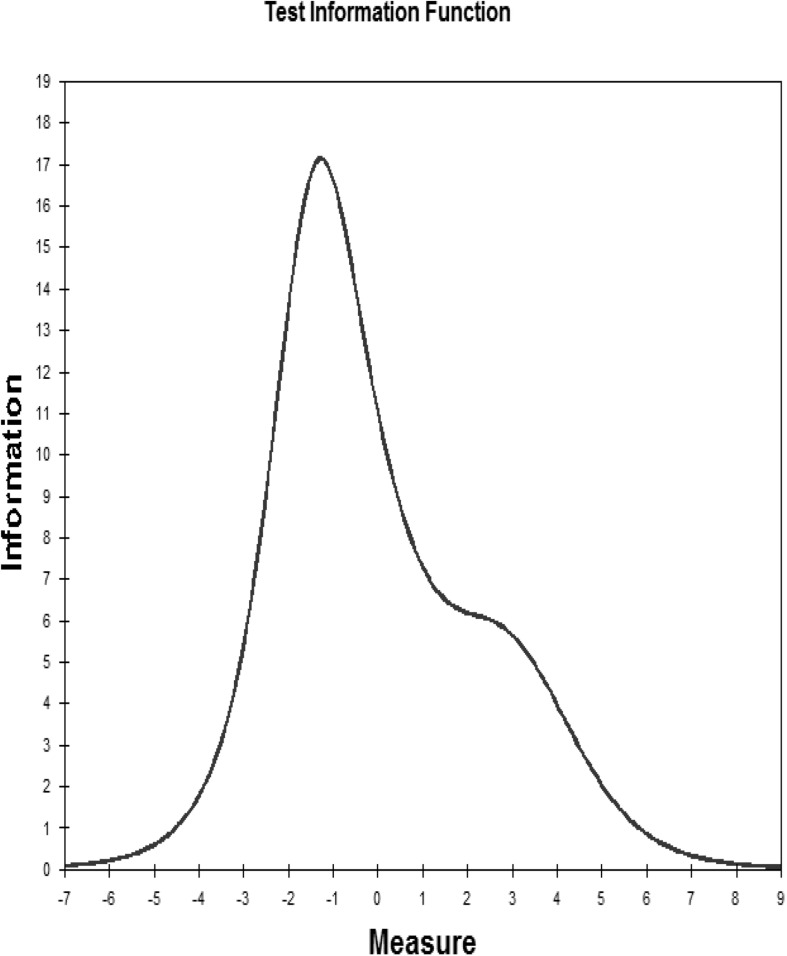
Test information function for the Myanmar version of the Medical Outcomes Study Social Support Survey.

The original MOS-SSS has a four-factor structure; that is, the factors of emotional/informational support (EMI; I3, I4, I8, I9, I13, I16, I17, and I19), tangible support (TAN; I2, I5, I12, and I15), affectionate support (AFF; I6, I10, and I20) and positive social interaction (POS; I7, I11, I14, and I18). As shown in Figure 1, the CFA showed and confirmed that the four-factor structure model exhibited satisfactory fit to our data [c2 (138) = 2.12, *p* = 0.00, RMSEA = 0.07, CFI = 0.95, and TLI = 0.94]. In the Rasch analysis, the unidimensionality assumption of the scale was supported by the PCA. That is, the residuals explained 54.50% (>50%) of the raw variance; the unexplained variance in the first contrast was 2.3 (<3.0) eigenvalue units. As shown in Table 2, the infit and outfit mean squares for each item ranged from 0.68 to 1.40; the item difficulty for each item ranged from −0.34 to 0.31; the DIF was not found when evaluated by gender and CD4 count level. No evidence of disordered thresholds was found in the category probability curves, as the category calibration increased in an orderly way (see Figure 2). We also calculated the person reliability (3.32) and person-separation index (0.92) in the analysis. Regarding the TIFs, both subscales gathered information most precisely when θ ranged from −2.0 to 0 (see Figure 3).

**TABLE 2:** The difficult, infit, outfit MNSQ, DIF, and corrected item-total correlation of 19 items.

Item	Item difficult^a^	Infit MNSQ	Outfit MNSQ	Corrected item-total correlation	DIF contrast by gender^b^	DIF contrast by CD4 count^c,d^	
(1) Help if confined to bed	0.12	0.93	0.91	0.78^†^	0.58	0.50	0.42
(2) Listen to you	0.04	1.00	0.93	0.76^†^	0.22	0.34	0.36
(3) Give you good advice	–0.11	0.90	0.91	0.79^†^	0.09	–0.52	–0.08
(4) Take to doctor	–0.14	1.00	0.86	0.74^†^	–0.22	0.59	0.24
(5) Show love and affection	–0.17	0.96	0.85	0.73^†^	–0.57	–0.07	–0.55
(6) Have a good time with	–0.04	1.02	1.17	0.74^†^	0.09	0.33	0.11
(7) Give you information	–0.21	0.94	0.85	0.80^†^	0.10	–0.14	0.06
(8) Confide in	–0.34	0.84	0.76	0.76^†^	0.09	–0.26	–0.27
(9) Hug you	0.27	0.91	0.86	0.79^†^	–0.12	0.28	0.02
(10) Get together for relaxation	0.31	1.03	1.07	0.77^†^	0.00	0.20	0.02
(11) Prepare meals	–0.03	1.01	0.98	0.75^†^	0.16	–0.15	0.15
(12) Give advice you really want	–0.12	0.68	0.68	0.83^†^	–0.19	–0.37	0.09
(13) Help get mind off things	0.18	0.99	0.99	0.79^†^	0.04	–0.20	–0.15
(14) Help with daily chores	0.09	1.36	1.40	0.64^†^	–0.25	0.08	–0.19
(15) Share worries with	0.03	0.96	1.00	0.77^†^	0.00	–0.80	–0.39
(16) Turn to for suggestions	–0.01	1.12	1.11	0.75^†^	0.00	–0.62	–0.09
(17) Do something enjoyable	0.30	1.12	1.04	0.77^†^	0.03	0.65	0.22
(18) Understand your problems	–0.19	0.99	0.89	0.76^†^	–0.18	–0.15	–0.14
(19) Love you	0.00	1.01	0.97	0.75^†^	0.07	0.39	0.14

*^†^*p* < 0.05; MNSQ, mean square; DIF, differential item functioning.*

*^*a*^Measured in logit; positive item logit indicates that the item requires a lower visual ability than the mean of the items and is an easier item, while a negative item logit indicates that the item requires a higher visual ability than the mean of the items and is a more difficult item.*

*^*b*^Male compared with female.*

*The DIF contrast by CD4 count in the following order:*

*^*c*^<200 cells/mm^3^ compared with 200–499 cells/mm^3^.*

*^*d*^<200 cells/mm^3^ compared with ≥500 cells/mm^3^.*

### Construct Validity

The item-scale correlations of the MOS-SSS-M were all greater than 0.3, including correlations that ranged from 0.80 to 0.89 for the tangible support scale, 0.84–0.88 for the affection scale, 0.79–0.91 for the emotional/informational scale, and 0.77–0.90 for the positive interaction scale. In addition, the item-other subscale correlations ranged from 0.57 to 0.73 and correlated higher by two standard errors with their subscales than with any of the other subscales. The concurrent validity of the MOS-SSS-M was confirmed with a negative correlation with the HIV stigma scale (*r* = −0.77, *p* < 0.001) and the PSSHIV (*r* = −0.53, *p* < 0.001).

### Internal Consistency

The Cronbach’s alpha for the MOS-SSS-M was 0.97 and the Cronbach’s alphas for the individual domains were 0.82–0.95. The corrected item-total correlation ranged from 0.64 to 0.83 (*p* < 0.05).

### Floor/Ceiling Effect

Among the study participants, 5.32% (5/94) and 1.55% (3/194) achieved the lowest possible score (19) and the highest possible score on the scale (95), respectively. The lowest or highest possible scores were both attained by less than 15% of the sample, indicating that there were no floor or ceiling effects of the MOS-SSS-M (Terwee et al., 2007).

### Description of the MOS-SSS-M

As shown in Table 3, the average total score of the MOS-SSS-M was (66.96 ± 16.34), and the lowest to the highest scores of the individual domains were on positive social interactions, affectionate support, tangible support, and emotional/informational support, respectively.

**TABLE 3:** The description of the overall and four domains of the MOS-SSS-M.

Domains	Item number	Range	Mean (SD)	Standardized score^a^ (%)
Emotional/informational support	8	8–40	28.55 (7.32)	71.37
Tangible support	4	4–20	14.10 (3.69)	70.51
Positive social interactions	4	4–20	13.78 (3.98)	68.90
Affectionate support	3	3–15	10.53 (2.74)	70.23
Total score	19	19–95	66.96 (16.34)	70.48

*^*a*^Standardized score = Mean/total score of domain × 100%.*

*MOS-SSS-M, the Myanmar version of the Medical Outcomes Study Social Support Survey.*

## Discussion

This paper is the first scale validation of the Myanmar version of MOS-SSS for the HIV-infected population. The MOS-SSS-M went through a multiphase process to ensure the rigorousness of the scale validation. The psychometric evaluation presented in this paper provides satisfactory cross-cultural, structural, and construct validities, as well as robust internal consistency and reliability. Floor and ceiling effects were not found. Therefore, the 19-item MOS-SSS-M can serve as a valid and reliable scale for understanding the level and types of social support in PLHIV in Myanmar.

Although the MOS-SSS differs as a construct across cultures (Yu et al., 2015), the CFA findings in this study yielded the same four-factor structure for the MOS-SSS-M as has been previously determined in other cultural contexts (e.g., American, Arabic, French, and Chinese; Robitaille et al., 2011; Thompson et al., 2014; Dafaalla et al., 2016). The findings indicate that the four-dimensional structure is appropriate for the Myanmar context and that the scale can be used to measure the level and categories of social support (emotional/informational, tangible, positive social interactions, and affectionate) obtained by PLHIV. The high convergent and discriminant validity of items also support the dimensionality of our measures (Sherbourne and Stewart, 1991).

In addition to traditional CTT, the structural validity of the MOS-SSS-M was also confirmed by Rasch analysis. Rasch analysis measures whether a respondent’s likelihood of having a given latent trait is assessed independently of the particular characteristics of the items administered, and is revealing of the trait and not simply the specific instrument. Thus, Rasch analysis makes up for the limitations of CTT (Kim et al., 2017). Our data support that the category rating scale of the MOS-SSS-M worked well, and the combination of a good person-separation index (>2) and person reliability (>0.8) suggest that the MOS-SSS-M has acceptable measurement precision. Also, this revised scale is sensitive to distinguishing both high and low levels of social support among PLHIV (Xu et al., 2018).

Regarding the TIF, when represented graphically, high TIF values are associated with low standard errors of measurement, and can thus indicate precision (Hambleton et al., 1991). The most precise information provided by the TIF for the MOS-SSS-M displays the precise and reliable measure of the low to middle levels of the scale. In addition, the Rasch measures also allow for the estimation of the equivalence of item calibrations across different samples and contexts (Kim et al., 2017). We examined how 19 items were used differently by PLHIV of different genders and with varying CD4 counts. The DIF findings showed that there were no gender and CD4 count differences in the item difficulty, which further supports the stability and validity of the MOS-SSS-M (Xu et al., 2018).

The significant negative correlations between the overall scale of the MOS-SSS-M and the HIV stigma scale and PSSHIV not only support concurrent validity of the MOS-SSS-M but also confirm that social support can function as a coping resource that buffers the effects of stressful events, stigma, and isolation (Kim et al., 2017). Furthermore, the Cronbach’s α of more than 0.7 indicates that the MOS-SSS -M has satisfactory internal consistency and reliability (Johnson et al., 2011).

Regarding the scores of individual subscales of the MOS-SSS-M, we found that our sample scored lower compared with incarcerated women (Kim and Mazza, 2014) and with PLHIVs in other cultural contexts (Abrefa-Gyan et al., 2016; Kim et al., 2017). The low levels of social support of PLHIV in Myanmar presented in the subscale of positive social interaction and affectionate support. In Myanmar, there is currently no welfare or job support for PLHIV and many face family or community rejection and stigma as a result of their HIV status, which thus limits their positive social activities and interactions (Tun et al., 2019). Compared to the often open expressing of love and emotions in Western culture, Burmese expressions of love in Myanmar are implicit and indirect; they seldom use the word “love” in their daily life, and—similar to other conservative Asian cultural contexts—such feelings are expressed indirectly or through acts of service (Yu et al., 2015).

### Implications

Evidence has consistently indicated that social support is a common facilitator of HIV prevention, testing, and treatment adherence. It also is an important predictor of QOL (Tun et al., 2019), especially in resource-limited countries such as Myanmar. The psychometric properties presented in this study suggest that the 19-item MOS-SSS-M can accurately measure the levels and types of social support (emotional/informational, tangible, positive social interactions, and affectionate) among PLHIV in Myanmar.

This scale could also facilitate the development of specific social support interventions or policies to help increase social support and improve the QOL of those diagnosed with HIV/AIDS in Myanmar, and it could be used to evaluate the effects of future interventions. Future research with more representative samples is needed to further examine the screening utility of this scale. It will also be important to determine the cut-off value for the MOS-SSS-M (the low, middle, and high levels of social support) to be able to compare the perceived social support of PLHIV in Myanmar with PLHIV globally. However, we found that the level of social support among PLHIV in Myanmar is inadequate and needs to be improved through culturally sensitive programs, especially in regard to positive social interactions and affectionate support.

### Limitations

This study has several limitations. First, our sample size was relatively small, and PLHIV in this study were much younger than 28.23-year—both of which impact the generalization of study findings. Second, some psychometric characteristics of the MOS-SSS-M could be assessed further, such as test-retest reliability. Third, we did not assess the sensitivity of the MOS-SSS-M. Therefore, future longitudinal or experimental studies are warranted. A further refinement of the scale using a larger representative sample will produce more stable parameter estimations and robust results. Fourth, since the MOS-SSS is a self-reported measure of social support, the measurement of whether the MOS-SSS accurately reflects actually received support requires further evaluation. Further studies such as focus groups or cognitive interviews may help evaluate how this population understands and formulates their responses to social support questions.

## Conclusion

The Myanmar version of the 19-item MOS-SSS (the MOS-SSS-M) with a four-factor structure was a sufficiently valid and reliable tool for assessing social support in the everyday lives of PLHIV in Myanmar. It can provide healthcare providers with an instrument for assessing the levels and categories of social support experienced by PLHIV in Myanmar. It also contributes to a better understanding of how social support operates within PLHIV in Myanmar and how it can affect them. Furthermore, the MOS-SSS-M can also facilitate the development of culturally sensitive social support interventions and, later, it can be used to evaluate the effects of such interventions.

## Data Availability Statement

The raw data supporting the conclusions of this article will be made available by the authors, without undue reservation.

## Ethics Statement

The studies involving human participants were reviewed and approved by this study was approved by the relevant institutional review boards (Number: #18-001769-CR-00001). The patients/participants provided their written informed consent to participate in this study.

## Author Contributions

W-TC: design, translation, and writing of the manuscript. FH: translation, data-analysis, and writing of the manuscript. SL, MT, and HO: data-collection and final approval. All authors contributed to the article and approved the submitted version.

## Conflict of Interest

The authors declare that the research was conducted in the absence of any commercial or financial relationships that could be construed as a potential conflict of interest.

## Publisher’s Note

All claims expressed in this article are solely those of the authors and do not necessarily represent those of their affiliated organizations, or those of the publisher, the editors and the reviewers. Any product that may be evaluated in this article, or claim that may be made by its manufacturer, is not guaranteed or endorsed by the publisher.
