# Supplementary material for: Translation, Adaption, and Psychometric Testing of the Myanmar Version of the Medical Outcomes Study Social Support Survey for People Living With HIV/AIDS
Source: Front Psychol. 2021 Sep 7;12:707142. doi: 10.3389/fpsyg.2021.707142 (PMC8452870; doi:10.3389/fpsyg.2021.707142)
Supplement: Supplementary file 1 [file Table_1.docx]

Appendix A. The Myanmar version of the Medical Outcomes Study Social Support Survey

| Item |
| --- |
| 2. အကယ်၍ သင်အိပ်ရာထဲလဲသွားပါက သင့်ကိုကူညီရန် တစ်ယောက်ယောက်ရှိသည်။  2. Someone to help you if you were confined to bed |
| 3. စကားပြောဖို့ လိုအပ်လာသည့်အခါ သင့်ကို နားထောင်ပေးရန် သင်အားကိုးရသူ တစ်ယောက်ယောက်ရှိသည်။.  3. Someone you can count on to listen to you when you need to talk. |
| 4. ဘေးကျပ်နံကျပ်ကာလတွင်. သင့်ကို အကြံကောင်းပေးနိုင်သူ တစ်ယောက်ယောက်ရှိသည်။.  4. Someone to give you good advice about a crisis |
| 5. လိုအပ်ပါက ဆရာဝန်ထံ သင့်ကို ခေါ်သွားရန် တစ်ယောက်ယောက်ရှိသည်။.  5. Someone to take you to the doctor if you needed it |
| 6. သင့်ကို မေတ္တာတရားနှင့် ချစ်ခင်မှုကို ပြသောသူ တစ်ယောက်ယောက်ရှိသည်။.  6. Someone who shows you love and affection. |
| 7. ပျော်ရွှင်ဖွယ်ကောင်းသော အရာများ ကို တူတူလုပ်ပေးနိုင်သောသူ.  7. Someone to have a good time with |
| 8. အခြေအနေတစ်ခုကို.သင်နားလည်လာအောင် သတင်းအချက်အလက်များ ပေးနိုင်သူ တစ်ယောက်ယောက်ရှိသည်။.  8. Someone to give you information to help you understand a situation. |
| 9. သင့်အကြောင်း (သို့) သင့်ပြဿနာများအကြောင်းကို ပြောရာတွင် စိတ်ချယုံကြည်ရသူ တစ်ယောက်ယောက်ရှိသည်။.  9. Someone to confide in or talk to about yourself or your problems. |
| 10. သင့်ကို ပွေ့ဖက်သူ တစ်ယောက်ယောက်ရှိသည်။.  10. Someone who hugs you. |
| 11. အနားယူအပန်းဖြေဖို့အတွက် အတူတူလုပ်ဆောင်ရန် တစ်ယောက်ယောက်ရှိသည်။.  11. Someone to get together with for relaxation. |
| 12. သင်ကိုယ်တိုင် အစားအသောက်များကို မပြုလုပ်နိုင်တော့သည့်. အခါ သင့်ကို လုပ်ကျွေးမည့်သူ တစ်ယောက်ယောက်ရှိသည်။.  12. Someone to prepare your meals if you were unable to do it yourself. |
| 13. သင်အမှန်တကယ်လိုချင်သော.အကြံဉာဏ်ကို ပေးနိုင်သူ တစ်ယောက်ယောက်ရှိသည်။.  13. Someone whose advice you really want. |
| 14. ပြဿနာများကို စိုးရိမ်ပူပန်ခြင်း တွေးတောခြင်း တို့မှ ရပ်တန့်အောင် ကူညီပေးနိုင်သူ တစ်ယောက်ယောက်ရှိသည်။.  14. Someone to do things with to help you get your mind off things. |
| 15. နာမကျန်းဖြစ်သောအခါ မိမိရဲ့ နေ့စဉ်အိမ်မှုကိစ္စများကို ကူညီလုပ်ကိုင်ပေးနိုင်သောသူ.  15. Someone to help with daily chores if you were sick. |
| 16. သင်၏ ပုဂ္ဂလိက စိုးရိမ်မှုများနှင့် အကြောက်တရားများကို မျှဝေနိုင်သူ တစ်ယောက်ယောက်ရှိသည်။.  16. Someone to share your most private worries and fears with. |
| 17. ကိုယ်ရေးကိုယ်တာ ပြဿနာများကို မည်ကဲ့သို့ ကိုင်တွယ်ဖြေရှင်းနိုင်ကြောင်း အကြံပြုချက်များပေးနိုင်သူ တစ်ယောက်ယောက်ရှိသည်။.  17. Someone to turn to for suggestions about how to deal with a personal problem. |
| 18. ကြည်နူးစရာအချိန်များကိုအတူတူ ကုန်ဆုံးနိုင်သူ တစ်ယောက်ယောက်ရှိသည်။.  18. Someone to do something enjoyable with. |
| 19. သင်၏ ပြဿနာများကို နားလည်သူ တစ်ယောက်ယောက် ရှိသည်။.  19. Someone who understands your problems. |
| 20. ကိုယ့်အပေါ် ချစ်ခင်ပြီး လိုလိုလားလားရှိသောသူ.  20. Someone to love and make you feel wanted. |
